# Working conditions and health status of 6,317 front line public health workers across five provinces in China during the COVID-19 epidemic: a cross-sectional study

**DOI:** 10.1186/s12889-020-10146-0

**Published:** 2021-01-09

**Authors:** Jinghua Li, Jingdong Xu, Huan Zhou, Hua You, Xiaohui Wang, Yan Li, Yuan Liang, Shan Li, Lina Ma, Jing Zeng, Huanle Cai, Jinzhao Xie, Chenghao Pan, Chun Hao, Stuart Gilmour, Joseph Tak-fai Lau, Yuantao Hao, Dong Roman Xu, Jing Gu

**Affiliations:** 1 School of Public Health, Sun Yat-sen University, No. 74, Zhongshan Second Road, Guangzhou, 510080 China; 2 Sun Yat-sen University Global Health Institute, School of Public Health and Institute of State Governance, Sun Yat-sen University, Guangzhou, 510080 China; 3 Hubei Province Center for Disease Control and Prevention, Wuhan, 430097 China; 4 West China School of Public Health and West China Fourth Hospital, Sichuan University, Chengdu, 610000 China; 5 School of Public Health, Nanjing Medical University, Nanjing, 210000 China; 6 School of Public Health, Lanzhou University, Lanzhou, 730000 China; 7 Guangzhou Center for Disease Control and Prevention, Guangzhou, 510440 China; 8 School of Public Health, Huazhong University of Science and Technology, Wuhan, 430074 China; 9 Zigong Center for Disease Control and Prevention, Zigong, 643000 China; 10 Graduate School of Public Health, St. Luke’s International University, Tokyo, 104-0045 Japan; 11 Centre for Health Behaviours Research, JC School of Public Health and Primary Care, The Chinese University of Hong Kong, Hong Kong, China; 12 Acacia Lab for Health Systems Strengthening and Department of Health Management, School of Health Management, School of Health Management, Southern Medical University, 1023 South Shatai Road, Guangzhou, 510515 China

**Keywords:** COVID-19, Front line public health workers, Mental health, Self-rated health, Working conditions, China

## Abstract

**Background:**

Public health workers at the Chinese Centre for Disease Control and Prevention (China CDC) and primary health care institutes (PHIs) were among the main workers who implemented prevention, control, and containment measures. However, their efforts and health status have not been well documented. We aimed to investigate the working conditions and health status of front line public health workers in China during the COVID-19 epidemic.

**Methods:**

Between 18 February and 1 March 2020, we conducted an online cross-sectional survey of 2,313 CDC workers and 4,004 PHI workers in five provinces across China experiencing different scales of COVID-19 epidemic. We surveyed all participants about their work conditions, roles, burdens, perceptions, mental health, and self-rated health using a self-constructed questionnaire and standardised measurements (i.e., Patient Health Questionnaire and General Anxiety Disorder scale). To examine the independent associations between working conditions and health outcomes, we used multivariate regression models controlling for potential confounders.

**Results:**

The prevalence of depression, anxiety, and poor self-rated health was 21.3, 19.0, and 9.8%, respectively, among public health workers (27.1, 20.6, and 15.0% among CDC workers and 17.5, 17.9, and 6.8% among PHI workers). The majority (71.6%) made immense efforts in both field and non-field work. Nearly 20.0% have worked all night for more than 3 days, and 45.3% had worked throughout the Chinese New Year holiday. Three risk factors and two protective factors were found to be independently associated with all three health outcomes in our final multivariate models: working all night for >3 days (multivariate odds ratio [ORm]=1.67~1.75, *p*<0.001), concerns about infection at work (ORm=1.46~1.89, *p*<0.001), perceived troubles at work (ORm=1.10~1.28, *p*<0.001), initiating COVID-19 prevention work after January 23 (ORm=0.78~0.82, *p*=0.002~0.008), and ability to persist for > 1 month at the current work intensity (ORm=0.44~0.55, *p*<0.001).

**Conclusions:**

Chinese public health workers made immense efforts and personal sacrifices to control the COVID-19 epidemic and faced the risk of mental health problems. Efforts are needed to improve the working conditions and health status of public health workers and thus maintain their morale and effectiveness during the fight against COVID-19.

**Supplementary Information:**

The online version contains supplementary material available at 10.1186/s12889-020-10146-0.

## Background

From 29 December 2019, coronavirus disease 2019 (COVID-19) [1] spread rapidly across China and has since become a global pandemic. As of 7 March 2020, a total of 80,695 confirmed cases and 3,097 deaths had been reported in China [2]. Despite some initial missteps, China implemented a series of draconian prevention and control measures that have been assessed favourably by the World Health Organization–China joint mission [3] and may have reduced the intensity of the local epidemic [4]. Since 27 January 2020, the number of daily new cases in China has been steadily declining [5]. In contrast, community outbreaks of COVID-19 have emerged quickly in many other countries, leading to strained health systems and containment efforts [6].

Given the lack of effective treatments and vaccines, preventive measures such as controlling the infection source, blocking transmission, and protecting susceptible populations are the most effective ways to contain the spread of COVID-19 [5, 7]. In response to the large-scale outbreak of COVID-19, China made an unprecedented effort to mobilise its public health workers through containment efforts. Although public health systems vary across countries [8–10], different countries may have similar needs related to COVID-19 prevention and control during this pandemic, and public health workers from different countries may face similar challenges. Therefore, other countries can learn from China’s experiences in terms of the types of public health workers who can be mobilised, the work roles and functions that these professionals can perform effectively, the challenges that they encounter, and, in particular, the heavy toll of the work on the workers’ physical and mental health. These lessons may enable other countries to better prepare their human resources in the fight against this pandemic.

During and after previous outbreaks and major public health emergencies, reports described the medical workers who joined the front lines to treat patients and documented their distress, difficulties, and health problems [11–14]. During earlier public health emergencies, such as the severe acute respiratory syndrome (SARS) outbreak, medical workers reported a high prevalence of depression and anxiety and perceptions of poor physical health [12, 15–17]. An increased workload, fear of infection resulting from patient care, inadequate training and equipment, and lack of support were identified as risk factors for poor mental and physical health [18–22]. Similar findings were reported in studies of medical workers who participated in the treatment of patients with Middle East respiratory syndrome (MERS) [23–25]. However, we have not identified any studies that focused on public health workers during outbreak control and prevention, despite their indispensable role in containing the epidemic.

China’s current public health system was established after the 2003 SARS epidemic [26]. This system comprises specialised institutions (e.g. disease prevention and control, maternal and child health, mental health) that provide technical guidance, and primary health care institutes (PHIs; e.g. community health centres/stations in urban areas and township health centres and village clinics in rural areas) that deliver public health services [26]. Structurally, the Centres for Disease Control and Prevention (CDC) in China have units at the national, provincial, municipal, and county/district levels, whereas PHIs operate at the town/subdistrict (i.e., community) level. During the COVID-19 epidemic in China, CDC units at all levels and PHIs undertook various tasks for prevention and control, including the development of technical instructions, epidemiological investigation of patients and close contacts, surveillance of high-risk populations, collection and examination of specimens, collection and reporting of data, delivery of health education and promotion, and administration of training [27].

It is important to document the working and health status of public health workers in China during the COVID-19 outbreak and to harness the experiences from this country, as this information can inform pandemic control efforts worldwide. In this study, we administered a large survey to front line public health workers across five provinces in China. We aimed to address the following key questions concerning the effective mobilisation and use of public health workers: (1) the roles and job functions (e.g. work tasks) of public health workers involved in epidemic containment efforts, (2) the working conditions and challenges associated with their work, (3) their perceptions (e.g. perceived support and troubles) related to COVID-19 and work, and (4) the factors associated with their mental and physical health (e.g. depression, anxiety, and self-rated health).

## Methods

### Study design

This cross-sectional study was conducted from 18 February to 1 March 2020. Data were collected from five provinces (Hubei, Guangdong, Sichuan, Jiangsu, and Gansu) that were purposely selected to cover different levels of epidemic severity as defined by the numbers of reported COVID-19 cases (as of 5 March 2020: 67,466, 1,351,539, 631, and 102 cases, respectively) and different regions of China (central, southern, western, eastern, and northern). Three to five cities were selected per province; within the selected cities, three to five districts/counties and five to 10 subdistricts/towns were further selected to represent both different outbreak levels and different regions. CDCs workers were investigated at the province, city, and district/county levels, and PHI workers were investigated at the subdistrict/town level. We targeted at least 5,000 public health workers and achieved a ratio of CDC to PHI workers of approximately 1:2.

### Participant recruitment

The eligible participants 1) were aged ≥ 18 years, 2) worked at a CDC unit or PHI in the selected locations during the study period, and 3) participated in COVID-19 control and prevention related work. Site investigators (e.g. CDC workers) in each province distributed the survey links through their WeChat/QQ working groups. WeChat and QQ are popular mobile phone communication/social networking applications used ubiquitously in workplace settings in China. All of the participants were informed of the background, aims, anonymous nature, and duration (approximately 8–12 minutes) of the survey. They were also informed that completing the questionnaire signified their informed consent. No compensation was provided to the participants. The study was approved by the ethics committee of the School of Public Health at Sun Yat-sen University (Reference no.: 2020-012).

### Measurements

#### Socio-demographic characteristics

We collected information on each participant’s age, sex, and job title, and whether they had children younger than 6 years old. Additionally, we collected information about the areas of routine work before the COVID-19 outbreak only from participants in Guangdong province during the pilot trial. We omitted this section from the survey administered to workers in other provinces after feedback from the pilot trial indicated that the questionnaire was too long.

#### COVID-19 control and prevention work related variables

We collected information from all of the participants about their work in terms of its content, their readiness, and its start time. Details of the variables are listed below.

1. Work content. The pre-set list of work content included 14 fieldwork questions covering topics such as face-to-face epidemiological investigations of the patients and close contacts, performance of epidemiological investigations by telephone or video calls, medical observation of the close contacts, collection and shipment of specimens, provision of health education, and performance of community-based investigations. It also addressed 11 non-field work topics, such as technical guideline preparation, data analysis and report writing, laboratory testing, comprehensive coordination and publicity, and technical training. The participants were asked to select the work that they performed from the pre-set list and fill in any other work content that was not included on the list.
2. Time spent in training on COVID-19. This was coded as none, 1–4 hours, 5–8 hours, 9–16 hours, or > 16 hours.
3. Knowledge of COVID-19 prevention and control. This was rated from 1, ‘adequate’ to 5, ‘very inadequate’.
4. Date when the participant began COVID-19 prevention and control work. In the data analysis, we selected the cut-off date of 23 January 2020 because this was both the date when Wuhan city was closed down and the day before Chinese New Year.
5. Epidemic severity in the worker’s province. This was rated from 1, ‘very low’, to 5, ‘very high’, according to the number of confirmed cases in each province.

#### Efforts and sacrifices

The participants were asked about their efforts and sacrifices during the outbreak. This information included 1) the number of days during which they worked all night, 2) whether they had worked throughout the Chinese New Year holiday, and 3) whether they had made family sacrifices such as not going home or sending their children to their own parents’ homes to avoid infecting family members.

*Perceptions related to COVID-19 and work* were also evaluated. One item was used to assess the workers’ concerns about being infected at work, and the responses were ranked from 1, ‘not concerned’, to 5, ‘very worried’. One item was used to assess how long the worker was able to maintain their current work intensity, and the responses were coded as 1–2 weeks, 3–4 weeks, 1–3 months, or > 3 months.

*Perceived support* and *perceived troubles at work* were measured using self-constructed items that were developed after discussions with CDC and PHI workers and among the research team. The *perceived support* scale comprised three items intended to measure the perceived support provided by colleagues, family, and society, and were rated on a Likert-type scale ranging from 1, ‘none’, to 5, ‘very much’. The three items showed acceptable internal consistency in this study (Cronbach’s alpha = 0.760). The *perceived troubles at work* scale comprised five items, which were rated on a 5-point Likert scale ranging from 1, ‘none’, to 5, ‘very much’. For example, the participants were asked how often they had been treated unfairly at work. In this study, the Cronbach’s alpha for perceived troubles was 0.842.

*Health outcomes* included overall health status, depression, and anxiety. The participants indicated their overall health by ranking their self-rated health status from 1, ‘very poor’, to 5, ‘very good’. This type of scale has been used widely in China and worldwide [28, 29]. The nine-item patient health questionnaire (PHQ-9) was used to assess the presence of depressive symptoms. The Chinese version of the PHQ-9 has been validated for the general population and showed good internal reliability [30]. The participants were asked to rate how often they had experienced depressive symptoms in the past 2 weeks by using a 4-point Likert scale ranging from 0, ‘none’, to 3, ‘nearly every day’. The total scores ranged from 0 to 27, with a higher score reflecting greater severity. A score ≥ 10 was classified as indicating a major depressive disorder. In this study, the Cronbach’s alpha value was 0.922.

The seven-item General Anxiety Disorder scale (GAD) was used to measure anxiety [31]. Each item was rated on a 4-point Likert scale ranging from 0, ‘never’, to 3, ‘often (almost every day)’. Scores at or above the cut-off value of 10 indicated a probable case of moderate anxiety disorder. In this study, the Cronbach’s alpha value was 0.937.

### Statistical analysis

Descriptive analysis was conducted to characterise the study. A chi-square test, *t*-test, and rank-sum test were used to investigate differences between the CDC and PHI workers. To explore the potential factors contributing to the three health outcomes, namely self-rated health, depression, and anxiety, three sets of logistic regression models were performed in parallel. First, bivariate logistic regression analyses were used to examine the associations between all variables of interest and the three outcomes. Next, adjusted logistic regression models were used to identify the associations between COVID-19-related variables (e.g., COVID-19 control and prevention work related variables, efforts and sacrifices during the outbreak, perceptions) and the three outcomes after controlling for potential confounders (sex, age, having children aged < 6 years, and job title). Finally, multivariate forward stepwise logistic regression models were fitted by using all COVID-19-related variables that were found to be significant in the univariate analysis as candidates for selection and by entering sociodemographic variables into the model. Unadjusted odds ratios (ORu) from the univariate logistic regression models, adjusted odds ratios (AOR) from the multiple logistic regression models, multivariate odds ratios (ORm) from the multivariate stepwise logistic regression, and their respective 95% confidence intervals (CIs) are reported. IBM SPSS Statistics 25 was used for the data analysis. A *p* value < 0.05 was considered to indicate statistical significance.

## Results

Of the 7,090 completed questionnaires, 528 (7.4%) did not pass the consistency checks and 245 (3.4%) did not include reports of any COVID-19-related work. We therefore performed a complete data analysis based on an effective sample size of 6,317 (89.1%).

### Socio-demographic characteristics of the participants

Of the 6,317 participants, 64.6% were female. The mean age was 38.7 years (SD = 9.43), and 66.9% were 30–49 years old. Additionally, 77.0% of the participants had an intermediate or junior job title, and 27.9% had children younger than 6 years old (Table 1). Compared with the PHI workers, the CDC workers included a larger proportion of participants who were male, younger, and held senior job titles.

**Table 1:** Descriptive statistics of public health workers during the COVID-19 epidemic (n, %)

Variables	All (*N* = 6317)	CDC employees (*n* = 2313)	PHI workers (*n* = 4004)	*p* value
**Socio-demographic characteristics**				
Sex (male)^c^	2238 (35.4)	987 (42.7)	1251 (31.2)	<0.001
Age groups (years)^d^				0.004
<29	1244 (19.7)	392 (16.9)	852 (21.3)	
30–39	2093 (33.1)	819 (35.4)	1274 (31.8)	
40–49	2132 (33.8)	748 (32.3)	1384 (34.6)	
>50	848 (13.4)	354 (15.3)	494 (12.3)	
Having children younger than 6 years (yes)^c^	1765 (27.9)	677 (29.3)	1088 (27.2)	0.074
Job title^c^				<0.001
Junior	2880 (45.6)	745 (32.2)	2135 (53.3)	
Intermediate	1984 (31.4)	800 (34.6)	1184 (29.6)	
Senior	712 (11.3)	504 (21.8)	208 (5.2)	
Others (e.g., volunteers)	741 (11.7)	264 (11.4)	477 (11.9)	
**COVID-19 control and prevention work related variables**				
Outbreak severity (by province)^a, d^				<0.001
Low	591 (9.4)	477 (20.6)	114 (2.8)	
Medium	3638 (57.6)	1224 (52.9)	2414 (60.3)	
High	830 (13.1)	282 (12.2)	548 (13.7)	
Very high	1258 (19.9)	330 (14.3)	928 (23.2)	
Work content^c^				<0.001
Involved in field work	1556 (24.6)	352 (15.2)	1204 (30.1)	
Involved in non-field work	235 (3.7)	149 (6.2)	86 (2.1)	
Involved in both field and non-field work	4526 (71.6)	1812 (78.3)	2714 (67.8)	
Duration of training on COVID-19^d^				<0.001
None	180 (2.8)	113 (4.9)	67 (1.7)	
1–4 hours	1128 (17.9)	411 (17.8)	717 (17.9)	
5–8 hours	876 (13.9)	339 (14.7)	537 (13.4)	
9–16 hours	939 (14.9)	350 (15.1)	589 (14.7)	
>16 hours	3194 (50.6)	1100 (47.6)	2094 (52.3)	
Level of knowledge of COVID-19 prevention and control^d^				<0.001
Adequate	975 (15.4)	275 (11.9)	700 (17.5)	
Relatively adequate	3700 (58.6)	1397 (60.4)	2303 (57.5)	
Average	1537 (24.3)	580 (25.1)	957 (23.9)	
Inadequate/very inadequate	105 (1.7)	61 (2.6)	44 (1.1)	
Start time of COVID-19 prevention and control work (after 23 January 2020)^c^	3613 (60.0)	1098 (50.1)	2515 (65.6)	<0.001
**Efforts and sacrifices during the outbreak**				
Number of days worked all night^d^				<0.001
0	3617 (57.3)	1073 (46.4)	2544 (63.5)	
1–3 days	1459 (23.1)	621 (26.8)	838 (20.9)	
>3 days	1241 (19.6)	619 (26.8)	622 (15.5)	
Worked throughout Chinese New Year (yes)^c^	2862 (45.3)	1313 (56.8)	1549 (38.7)	<0.001
To avoid infecting family members, chose not to live at home (yes)^c^	878 (13.9)	305 (13.2)	573 (14.3)	0.213
To avoid infecting family members, sent children to parents’ homes (yes)^c^	935 (14.8)	338 (14.6)	597 (14.9)	0.749
**Perceptions**				
Worried about being infected at work^d^				<0.001
None/mild	2461 (39.0)	1023 (44.2)	1438 (35.9)	
Medium	2264 (35.8)	795 (34.4)	1469 (36.7)	
Very worried	1592 (25.2)	495 (21.4)	1097 (27.4)	
Perceived ability to maintain current work intensity for ≥1 month^d^	2315 (36.6)	750 (32.4)	1565 (39.1)	<0.001
Perceived support from colleagues^d^				0.098
None/low	383 (6.1)	143 (6.2)	240 (6.0)	
Medium	2448 (38.8)	858 (37.1)	1590 (39.7)	
High/very high	3486 (55.2)	1312 (56.7)	2174 (54.3)	
Perceived support from family^d^				<0.001
None/low	122 (1.9)	48 (2.1)	74 (1.8)	
Medium	1334 (21.1)	410 (17.7)	924 (23.1)	
High/very high	4861 (77.0)	1855 (80.2)	3006 (75.1)	
Perceived support from society^d^				0.014
None/low	757 (12.0)	323 (14.0)	434 (10.8)	
Medium	2779 (44.0)	999 (43.2)	1780 (44.5)	
High/very high	2781 (44.0)	991 (42.8)	1790 (44.7)	
Total score of perceived support^b^	10.99 ± 1.95	11.02 ± 1.91	10.98 ± 1.97	0.360
Perceived troubles at work				
Your work was not understood^d^				0.002
None/rare	2671 (42.3)	918 (39.7)	1753 (43.8)	
Sometimes	2490 (39.4)	945 (40.9)	1545 (38.6)	
Much/very much	1156 (18.3)	450 (19.5)	706 (17.6)	
You have been treated unfairly at work^d^				<0.001
None/rare	3471 (54.9)	1191 (51.5)	2280 (56.9)	
Sometimes	1997 (31.6)	753 (32.5)	1244 (31.1)	
Much/very much	849 (13.4)	369 (16.0)	480 (12.0)	
You felt wronged at work^d^				<0.001
None/rare	3022 (47.8)	1030 (44.5)	1992 (49.8)	
Sometimes	2245 (35.5)	848 (36.7)	1397 (34.9)	
Much/very much	1050 (16.6)	435 (18.8)	615 (15.4)	
Your family cannot understand your efforts^d^				0.326
None/rare	4976 (78.8)	1837 (79.4)	3139 (78.4)	
Sometimes	1076 (17.0)	384 (16.6)	692 (17.3)	
Much/very much	265 (4.2)	92 (4.0)	173 (4.3)	
You worried about routine works besides the COVID-19 epidemic^d^				<0.001
None/rare	2730 (43.2)	1085 (46.9)	1645 (41.1)	
Sometimes	2527 (40.0)	869 (37.6)	1658 (41.4)	
Much/very much	1060 (16.8)	359 (15.5)	701 (17.5)	
Total score of perceived troubles at work	12.25 ± 3.74	12.39 ± 3.82	12.17 ± 3.69	0.023
**Mental health status and self-perceived health**				
PHQ-9^b^	5.94 ± 5.59			
Depression (yes)^c^	1034 (21.3)	521 (27.1)	513 (17.5)	<0.001
GAD-7^b^	5.69 ± 5.07			
Anxiety (yes)^c^	920 (19.0)	396 (20.6)	524 (17.9)	<0.001
Self-perceived health^d^				<0.001
Very good	974 (15.4)	199 (8.6)	775 (19.4)	
Good	2504 (39.6)	841 (36.4)	1663 (41.5)	
Average	2221 (35.2)	926 (40.0)	1295 (32.3)	
Poor	555 (8.8)	303 (13.1)	252 (6.3)	
Very poor	63 (1.0)	44 (1.9)	19 (0.5)	

*Abbreviations*: COVID-19 coronavirus disease 2019, *PHQ-9* 9-item Patient Health Questionnaire, *GAD-7* seven-item Generalized Anxiety Disorder scale

^a^ Epidemic severity (by province): 1. Very low (0–19); 2. Low (20–199); 3. Medium (200–699); 4. High (700–9,999); 5. Very high (> 10,000). Values represent the total number of confirmed cases in each province by 25 February 2020.

^b^ The *t*-test was used.

^c^ The chi-square test was used.

^d^ The rank-sum test was used.

### COVID-19 control and prevention work related variables

As shown in Table 1, 19.9% of the participants were located in a province with severe epidemic conditions (e.g. Hubei); these workers accounted for 14.3% of the CDC workers and 23.2% of the PHI workers, and this difference was significant (*p* < 0.001). Nearly half (49.9) of the CDC workers had begun performing COVID-19 prevention work before 23 January 2020, compared with 34.4% of the PHI workers (*p* < 0.001). Before 20 January 2020, 22.0% of the CDC workers and 9.0% of the PHI workers were performing COVID-19 control and prevention work, and these proportions had increased to 87.0 and 78.0%, respectively, by 27 January. Both the CDC and PHI workers self-reported that they had received training and had sufficient knowledge about COVID-19. Fewer than 2.0% of the participants reported having inadequate knowledge (2.6% of the CDC workers versus 1.1% of PHI workers), and more than half had received > 16 hours of training (47.6% of the CDC workers versus 52.3% of the PHI workers).

The majority of the workers (78.3% of the CDC workers versus 67.8% of the PHI workers, *p* < 0.001) participated in both field and non-field work (Table 1). Detailed information on the work content is presented in Table S1. Notably, 22.8% of the public health workers participated in face-to-face epidemiological investigations of patients (17.8% of the CDC workers versus 25.8% of the PHI workers, *p* < 0.001), while 22.8% participated in medical observations of close contacts (10.2% of the CDC workers versus 30.1% of the PHI workers, *p* < 0.001). Additionally, 22.7% of the CDC workers and 52.2% of the PHI workers provided health education, and 26.0% of the CDC workers and 9.0% of the PHI workers were involved in epidemiological report writing.

In Guangdong province (Table S2), 88.6% of CDC workers involved in COVID-19 prevention work were focused on public health, compared with 37.0% of PHI workers (*p* < 0.001). Specifically, 63.0% of PHI workers engaged in COVID-19 prevention work cited that they routinely worked as clinicians (27.6%), nurses (26.6%), pharmacists (5.1%), and clinical technicians (3.7%). The majority of CDC workers were routinely employed in fields with a public health concentration, such as infectious disease prevention and control (16.1%), non-communicable disease prevention and control (20.1%), health education (6.1%), and health inspection (9.3%).

### Efforts and sacrifices during the outbreak

Regarding effort, as presented in Table 1, significantly more of the CDC workers (26.8%) reported that they had worked all night for > 3 days, compared with the PHI workers (15.5%, *p* < 0.001). Additionally, 56.8% of the CDC workers and 38.7% of the PHI workers had worked throughout the Chinese New Year (*p* < 0.001). Regarding sacrifices, no significant differences were observed between the CDC workers and PHI workers. To avoid infecting family members, 13.9% of the participants had chosen not to live at home and 14.8% had sent their children to their own parents’ homes.

### Perceptions

Compared with the CDC workers (55.8%), more of the PHI workers (64.1%, *p* < 0.001) reported moderate to high levels of concern about infection at work. The majority of the workers (88.0–98.1%, Table 1) perceived a medium or high level of support from their colleagues, family, and society. There were no significant differences in the total scores pertaining to support between the CDC and PHI workers (mean, 11.02 versus 10.98, *p* = 0.360). The CDC workers perceived a higher level of troubles at work than the PHI workers did (mean, 12.39 versus 12.17, *p* = 0.023). For example, 48.5% of the CDC workers and 43.1% of the PHI workers reported that they had been treated unfairly at work (*p* < 0.001, Table 1), and 53.1% of the CDC workers and 58.9% of the PHI workers worried about their routine work in addition to their COVID-19 prevention work (*p* < 0.001). Descriptive statistics corresponding to the public health workers’ perceptions are shown in Table 1.

### Self-rated health status and prevalence of mental health problems

As shown in Table 1, fewer than 10.0% of the participants reported poor/very poor self-rated health (15.0% of the CDC workers versus 6.8% of the PHI workers, *p* < 0.001). The prevalence of probable depression was 27.1% among the CDC workers versus 17.5% among the PHI workers (*p* < 0.001). The prevalence of anxiety was 20.6% among the CDC workers versus 17.9% among the PHI workers (*p* < 0.001).

### Factors associated with depression, anxiety, and poor self-rated health

The associations between background variables and the three health outcomes are presented in Table 2. Significant socio-demographic variables associated with depression included being female (ORu = 1.29), having children younger than 6 years (ORu = 1.41), and being older than 30 years (ORu = 0.63). Only one significant socio-demographic variable, namely having children younger than 6 years old (ORu = 1.39), was found to be associated with anxiety. Women (ORu = 1.13), participants with children younger than 6 years old (ORu = 1.12), and participants with an intermediate or senior job title (ORu = 1.26) were more likely to self-rate their health as poor. Compared with the participants from other provinces, those from Hubei province reported a higher level of anxiety but not of depression or poor self-rated health. After adjustment for socio-demographic variables, 10 of the 14 factors of interest were significantly associated with all three health outcomes (i.e. depression, anxiety, and poor self-rated health), including three protective factors and seven risk factors (Table 2).

**Table 2:** Associations between mental health status/self-perceived health status and background variables

Variables	Depression			Anxiety			Poor self-perceived health		
	Row (%)	ORu	AOR (95% CI)	Row (%)	ORu	AOR (95% CI)	Row (%)	ORu	AOR (95% CI)
**Socio-demographic characteristics**									
Sex									
Male	18.7	1.00	NA	17.8	1.00	NA	43.0	1.00	NA
Female	22.9	1.29^**^	NA	19.6	1.13	NA	46.0	1.13^*^	NA
Age group (years)									
<30	28.0	1.00	NA	20.8	1.00	NA	44.0	1.00	NA
≥30	19.6	0.63^***^	NA	18.5	0.86	NA	45.2	1.05	NA
Having children under 6 years									
No	19.8	1.00	NA	17.6	1.00	NA	44.2	1.00	NA
Yes	25.8	1.41^***^	NA	22.9	1.39^***^	NA	47.0	1.12^*^	NA
Job title									
Junior	20.9	1.00	NA	19.4	1.00	NA	42.5	1.00	NA
Intermediate/senior	21.4	1.03	NA	18.4	0.94	NA	48.3	1.26^***^	NA
Others (e.g., volunteers)	22.7	1.11	NA	19.1	0.98	NA	42.0	0.98	NA
**COVID-19 control and prevention work-related variables**									
Outbreak severity (by province)^**1**^									
Hubei	21.0	1.00	1.00	20.6	1.00	1.00	44.3	1.00	1.00
Other provinces	21.4	1.03	0.95 (0.81,1.11)	18.4	0.87	0.83 (0.70,0.98)*	45.1	1.03	1.01 (0.89,1.14)
Work content									
Involved in field work only	19.2	1.00	1.00	17.3	1.00	1.00	38.1	1.00	1.00
Involved in non-field work only	29.5	1.76^**^	1.57 (1.10,2.23)*	16.4	0.94	0.88 (0.58,1.35)	51.1	1.70^***^	1.64 (1.24,2.16)^***^
Involved in both work types	21.5	1.16	1.25 (1.05,1.49)*	19.6	1.16	1.21 (1.01,1.45)*	47.0	1.44^***^	1.46 (1.29,1.64)^***^
Institution									
CDC	27.1	1.00	1.00	20.6	1.00	1.00	55.0	1.00	1.00
PHI	17.5	0.57^***^	0.57 (0.49,0.66)^***^	17.9	0.84^*^	0.84 (0.72,0.97)^*^	39.1	0.53^***^	0.53 (0.47,0.59)^***^
Received training on COVID-19									
None	28.9	1.00	1.00	22.5	1.00	1.00	56.7	1.00	1.00
Yes	21.1	0.66^*^	0.70 (0.48,1.01)	18.9	0.80	0.83 (0.56,1.24)	44.6	0.62^**^	0.62 (0.46,0.84)^**^
Had sufficient knowledge of COVID-19 prevention and control									
Adequate/relatively adequate	19.2	1.00	1.00	17.6	1.00	1.00	39.8	1.00	1.00
Average	25.7	1.46^***^	1.41 (1.21,1.65)^***^	21.7	1.30^**^	1.27 (1.08,1.49)^**^	58.6	2.14^***^	2.19 (1.94,2.46)^***^
Inadequate/very inadequate	43.5	3.23^***^	3.16 (2.07,4.84)^***^	37.0	2.75^***^	2.68 (1.74,4.14)^***^	76.2	4.85^***^	4.88 (3.10,7.69)^***^
Start time of COVID-19 prevention and control work									
Before 23 January 2020	23.3	1.00	1.00	21.5	1.00	1.00	50.8	1.00	1.00
On or after 23 January 2020	19.4	0.79^**^	0.71 (0.62,0.83)^***^	16.9	0.74^***^	0.70 (0.60,0.82)^***^	40.6	0.66^***^	0.64 (0.58,0.72)^***^
**Efforts and sacrifices during the outbreak**									
Number of days worked all night									
0	17.4	1.00	1.00	14.6	1.00	1.00	39.3	1.00	1.00
1–3	23.3	1.45^***^	1.61 (1.35,1.91)^***^	21.5	1.60^***^	1.71 (1.43,2.05)^***^	50.1	1.55^***^	1.65 (1.46,1.88)^***^
>3	28.0	1.85^***^	2.14 (1.80,2.55)^***^	25.9	2.05^***^	2.25 (1.88,2.70)^***^	55.3	1.91^***^	2.11 (1.84,2.41)^***^
Worked throughout Chinese New Year									
No	20.5	1.00	1.00	17.3	1.00	1.00	39.7	1.00	1.00
Yes	22.0	1.09	1.25 (1.08,1.44)^**^	20.4	1.23^**^	1.31 (1.13,1.52)^***^	51.3	1.61^***^	1.73 (1.56,1.92)^***^
To avoid infecting family members, chose not to live at home									
No	20.0	1.00	1.00	18.2	1.00	1.00	43.6	1.00	1.00
Yes	28.2	1.57^***^	1.53 (1.28,1.83)^***^	23.2	1.36^**^	1.36 (1.13,1.64)^**^	53.3	1.48^***^	1.53 (1.32,1.77)^***^
To avoid infecting family members, sent children to parents’ homes.									
No	20.7	1.00	1.00	18.1	1.00	1.00	44.4	1.00	1.00
Yes	24.8	1.26^**^	1.14 (0.94,1.38)	23.8	1.41^***^	1.29 (1.06,1.57)^*^	47.9	1.15^*^	1.11 (0.96,1.29)
**Perceptions**									
Worry about being infected at work									
None/mild	13.0	1.00	1.00	9.7	1.00	1.00	35.1	1.00	1.00
Medium/very worried	26.9	2.46^***^	2.42 (2.07,2.83)^***^	25.3	3.16^***^	3.12 (2.63,3.71)^***^	51.2	1.94^***^	1.92 (1.73,2.14)^***^
Perceived ability to maintain current intensity of work									
<1 month	26.7	1.00	1.00	23.4	1.00	1.00	54.1	1.00	1.00
≥1 month	11.5	0.36^***^	0.36 (0.31,0.43)^***^	11.0	0.41^***^	0.40 (0.34,0.48)^***^	29.2	0.35^***^	0.35 (0.32,0.40)^***^
Perceived support		0.93^***^	0.94 (0.90,0.97)^***^		0.98	0.98 (0.95,1.02)		0.89^***^	0.89 (0.87,0.92)^***^
Perceived troubles at work		1.29^***^	1.29 (1.26,1.32)^***^		1.32^***^	1.32 (1.29,1.35)^***^		1.16^***^	1.16 (1.14,1.18)^***^

*Abbreviations*: *ORu* odds ratio of univariate analysis, *AOR* odds ratio adjusted for sociodemographic variables (sex, age, having a child <6 years of age, job title), *CI* confidence interval, *COVID-19* coronavirus disease 2019, *CDC* Centre for Disease Control and Prevention, *PHI* primary health care institute

^*^ *p* < 0.05; ^**^p < 0 .01; ^***^p < 0.001; ORs and 95% CIs with *p* values < 0.05 are in bold.

In our final multivariate models (Table 3), three risk factors and two protective factors were found to be independently associated with all three health outcomes. The three risk factors included a) having worked all night for > 3 days (ORm = 1.67, 1.75 and 1.67 in relation to depression, anxiety, and poor perceived health, respectively), b) worry about being infected at work (ORm = 1.49, 1.89, and 1.46, respectively), and c) perceived troubles at work (ORm = 1.26, 1.28, and 1.10, respectively). The two protective factors included a COVID-19 prevention work start date after 23 January (ORm = 0.80, 0.78, and 0.82 in relation to depression, anxiety, and poor perceived health, respectively) and the ability to maintain the current work intensity for more than 1 month (ORm = 0.48, 0.55, and 0.44, respectively). Involvement only in non-field work was positively associated with depression (ORm = 1.89, *p* = 0.002) and poor self-rated health (ORm = 1.74, *p* < 0.001). Perceived support (ORm = 0.94, *p* < 0.001) was negatively associated with poor self-rated health.

**Table 3:** Associations between mental health status/self-perceived health status and background variables (multivariate stepwise logistic regression)^a^

Variables	Depression		Anxiety		Poor self-perceived health	
	ORm (95% CI)	*p* value	ORm (95% CI)	*p* value	ORm (95% CI)	*p* value
**COVID-19 control and prevention work related variables**						
Work content						
Involved in field work only	1.00		N.S.		1.00	
Involved in non-field work only	**1.89 (1.27,2.82)**	**0.002**	N.S.		**1.74 (1.28,2.36)**	**<0.001**
Involved in both work types	1.04 (0.85,1.27)	0.686	N.S.		**1.25 (1.09,1.43)**	**0.001**
Level of knowledge of COVID-19 prevention and control						
Adequate/relatively adequate	1.00		N.S.		1.00	
Average	1.18 (0.99,1.41)	0.064	N.S.		**2.02 (1.77,2.30)**	**<0.001**
Inadequate/very inadequate	**2.18 (1.33,3.59)**	**0.002**	N.S.		**3.64 (2.23,5.92)**	**<0.001**
Start time of COVID-19 prevention and control work (on or after 23 January 2020)	**0.80 (0.68,0.94)**	**0.008**	**0.78 (0.66,0.92)**	**0.004**	**0.82 (0.72,0.93)**	**0.002**
**Efforts and sacrifices during the outbreak**						
Number of days worked all night						
0	1.00		1.00		1.00	
1–3	**1.42 (1.16,1.72)**	**0.001**	**1.51 (1.23,1.85)**	**<0.001**	**1.51 (1.32,1.74)**	**<0.001**
>3	**1.67 (1.37,2.05)**	**<0.001**	**1.75 (1.42,2.16)**	**<0.001**	**1.67 (1.43,1.96)**	**<0.001**
Worked throughout Chinese New Year (yes)	N.S.		N.S.		**1.40 (1.23,1.59)**	**<0.001**
To avoid infecting family members, chose not to live at home (yes)	N.S.		N.S.		**1.23 (1.04,1.45)**	**0.014**
**Perceptions**						
Worry about being infected at work (medium/very worried)	**1.49 (1.25,1.78)**	**<0.001**	**1.89 (1.56,2.28)**	**<0.001**	**1.46 (1.30,1.65)**	**<0.001**
Perceived ability to maintain current intensity of work (≥1 month)	**0.48 (0.40,0.58)**	**<0.001**	**0.55 (0.45,0.67)**	**<0.001**	**0.44 (0.39,0.49)**	**<0.001**
Perceived support	N.S.		N.S.		**0.94 (0.92,0.97)**	**<0.001**
Perceived troubles at work	**1.26 (1.23,1.29)**	**<0.001**	**1.28 (1.25,1.31)**	**<0.001**	**1.10 (1.08,1.12)**	**<0.001**

*Abbreviations*: *ORm* multivariate odds ratio, *CI* confidence interval, *COVID-19* coronavirus disease 2019, *N.S.* non-significant

^a^ Variables that were significant in the univariate analyses (Table 2) were used as candidates in the forward stepwise models after adjusting for sociodemographic variables (sex, age, having a child < 6 years of age, job title).

Variables that were non-significant for all outcome variables, including training on COVID-19 and sending children to parents’ home to avoid infecting family members, are not included in this table.

Variables with *p* < 0.05 are in bold.

## Discussion

This is one of the few studies to provide timely documentation of the working status and health-related conditions of public health workers during a new and emerging infectious disease pandemic. We found that the surveyed public health workers made huge efforts and personal or family sacrifices during the COVID-19 control and prevention response, and 27.1 and 20.6% of the workers reported experiencing depression and anxiety, respectively. We further determined that the public health workers’ employment conditions were associated with their health status.

Our survey suggested that China efficiently and effectively mobilised its public health workforce in preparation for the epidemic, and that workers responded rapidly. Approximately one in five CDC workers had already been assigned to the epidemic control programme before 20 January 2020, although only the surveyed municipalities in Hubei province had reported cases by that date. In the following week, which coincided with the Chinese New Year, the CDC workers’ participation rate increased substantially, to nearly 90%. As community-level preventive work is coordinated by the CDC, PHI workers began to participate at a slightly later time point. Overall, the public health workers we surveyed had made huge efforts to control the epidemic: approximately half of them had worked throughout the Chinese New Year, which is the most important holiday in China, and one in five had worked all night for > 3 days at the time of this survey. Again, CDC workers were most likely to have worked throughout the holiday period and to have worked more full nights.

The prompt responses and great efforts of public health workers, together with other important strategies implemented by the Chinese government, probably hastened the decline of the epidemic in China. Through modelling studies, researchers have shown that highly effective contact tracing and case isolation can control a new outbreak of COVID-19 within 3 months [32]. Such work suggests the importance of traditional epidemiological measures such as infected case identification and isolation, contact tracing, and health education on protective behaviours. Through our survey, we confirmed that Chinese public health workers were actively engaged in such traditional epidemiological roles and thus most likely contributed significantly to the rapid control of the COVID-19 outbreak in China.

However, our survey results also indicated a lack of appreciation by the affected communities regarding the work performed by the public health workers [33]. Our data showed that although the workers received a high level of support from family and colleagues, they received relatively little support from society. In contrast with the work of physicians and nurses, the work performed by public health workers is not widely understood and respected by the public [33]. During the epidemic, the types of prevention and control work performed by the public health workers, such as the isolation of close contacts (at home or designated hotels) and home inspections, were very likely to elicit negative emotions and even objections in communities. Such negative emotions and conflicts not only hindered preventive work but also negatively impacted the workers’ mental health. In our analysis, we found that the public health workers’ perceived employment troubles and difficulties (e.g. unfair treatment, concerns about routine work) were independent risk factors for mental health problems and poor self-assessed health after controlling for potential confounders. Therefore, to ensure both epidemic control and public health workers’ well-being, it is extremely important to increase our understanding of the importance of public health work both in normal times and at the beginning of an epidemic.

The mental health status of public health workers during the COVID-19 pandemic warrants attention. Given the lack of similar previous surveys of public health workers, we can only compare our results with those of surveys of medical workers (a proxy for public health workers). In our study, the prevalence rates of self-reported depression (21.3%) and anxiety (19.0%) were substantially higher than those reported in a study of hospital medical staff performing daily work in China (18.3 and 14.7%, respectively), despite using the same measurements and cut-off values [34]. Furthermore, in our study, a larger proportion of CDC workers reported mental health problems and poor physical health compared with PHI workers. The former group might bear more responsibility for regional disease prevention [26] and perform longer work shifts. In studies of medical workers during the SARS epidemic, 38.5% of hospital workers who treated SARS patients developed symptomatic depression [12]. During the MERS epidemic, two thirds of medical workers reported psychological problems [24]. Although the findings of those studies are not comparable because different measurements were used, front line medical doctors and nurses who treat patients may have a higher prevalence of mental health problems than public health workers. In addition to immediate personal welfare concerns, health workers may find that mental health problems affect their work [35]. Accordingly, mental health care is essential for these front line health workers.

We additionally found that longer work durations and worries about infection were associated with both the participants’ mental health and their self-rated health status, similar to the findings of other studies [19, 20]. Participants who were involved in non-field work were more likely than others to have probable depression and poor self-reported health. Time-consuming paperwork, data analysis, and laboratory work may add to people’s physical and mental health burdens, especially when performed under emergency conditions [36]. We further found that the participants involved in non-field work reported longer working hours. Our findings suggest that the health status of public health workers in offices or laboratories should not be neglected in the area of outbreak control.

Our survey results also suggest that child care support could reduce public health workers’ stress levels and improve their commitment to work, and this measure is highly warranted in the future. In an earlier study, child care support was reported as one of the top support needs of health care employees working during a disastrous event [37]. Approximately one third of the surveyed public health workers in our study were parents of young children (28%). For health care workers, child care obligations can make it harder to commit to front line work during a catastrophic event [38]. An earlier survey of health care workers who participated in the SARS epidemic response revealed that living with children was significantly associated with an increased level of concern for personal or family health [39]. Together, the high volume of work and concerns about personal and family health can lead to burnout [40]. Other countries must consider child care support measures in their preparations for epidemic/pandemic control if they aim to ensure an effective public health workforce.

An epidemic outbreak is likely to place constraints on health workforces. In our study, 63% of the PHI workers who participated in COVID-19 prevention efforts did not routinely work in public health fields (27.6% clinicians, 26.6% nurses, and 5.1% pharmacists) had been recruited into public health work. Rapid training enabled them to effectively take up their new roles in community-based prevention during the COVID-19 outbreak. Furthermore, we observed training rates as high as 98%, and most of this training was conducted using Web-based online modules. Meanwhile, the smartphone-based online survey is widely used in epidemiological surveys. This suggests that mobile technologies could enable more efficient outbreak prevention strategies. China’s experiences suggest that successful task-shifting in public health work is possible during an epidemic crisis, particularly when timely training and technology are used concurrently.

This study has several limitations. First, selection bias may exist, as this study used a non-random sample. Although our stratified sampling strategy covered provinces that were located in different regions of China and affected by the epidemic to different degrees, the findings of this study might not be generalisable to all public health workers in China. Second, reporting bias may exist because of the nature of self-reported data. As the current study used an online questionnaire, people who were less skilled or did not have access to the Internet and/or a smartphone may have been underrepresented. Furthermore, people who are experiencing work strain may be less likely to engage with social networks such as WeChat groups. Consequently, we may not have recruited the people most at risk for our sample. We also note that we used online surveys, and therefore the response rate may not have been high. Third, the measurements of perceived support and perceived troubles at work were self-constructed and have not been validated, although both received high Cronbach’s alpha values for this study. Fourth, we only included participants in Guangdong province in our investigation of the areas of routine work. Although the national prevention strategy may lead to similar answers in other provinces, this finding might not be generalisable to the other provinces in our study. Fifth, we could not derive any conclusive causal relationships from a cross-sectional design.

Despite those limitations, our study provides a rare and focused evaluation of CDC and PHI workers. It is critical to understand the experiences of these workers, as they serve as the backbone of pandemic control efforts. We examined not only their physical health but also their mental health and their ability to maintain their workloads, as these are all crucial components of an effective pandemic response. The survey was conducted in a timely manner, with the aims of reducing recall bias and providing urgently needed information. Lastly, although we did not use a random sample, we did reach a large number of workers within a short period of time. Our results suggest that the government should include components to provide mental and physical support to PHI workers in their pandemic preparedness plans. Furthermore, although pandemic response work can be exhausting and is associated with mental and physical distress, our findings demonstrate the efficacy of non-public health workers in this context. We additionally provide evidence that rapidly training these workers to step into pandemic prevention roles can be an effective and valid strategy for expanding the pool of public health workers during a crisis and thus improving pandemic responsiveness while protecting staff well-being. Finally, it would be useful to improve the coordination and integration of public health workers and PHI workers.

## Conclusions

With this study, we are among the first to document the working situations and health status of a large and relatively representative sample of public health workers during the COVID-19 outbreak in China. The various public health systems in all countries have been challenged by the rapid global spread of the COVID-19 pandemic. It is important to provide the necessary support to public health workers to ensure their health and working conditions, as these factors are a key issue in epidemic control.

## Supplementary Information

**Additional file 1: Table S1**. Work contents during the COVID-19 epidemic. **Table S2**. Areas of routine work of public health workers in Guangdong province during the COVID-19 epidemic.

**Additional file 2.**

## Data Availability

All of the data generated or analysed during this study are included in this published article.

## Abbreviations

PHIs: Primary health care institutes
COVID-19: Coronavirus disease 2019
WHO: World Health Organization
CDC: Centers for Disease Control and Prevention
GAD: General Anxiety Disorder scale
ORu: Unadjusted odds ratio
AOR: Adjusted odds ratio
ORm: Multivariate odds ratio
CIs: Confidence intervals

## References

[1] National Health Commission of the People’s Republic of China, National Administration of Traditional Chinesse Medicine of the PRC. Guidance for corona virus disease 2019: prevention, control, diagnosis and management China: People’s Medical Publishing House,. WHO collaborating centre for health information and publishing; 2020.

[2] National Health Commission of the People’s Republic of China (2020). Updates on COVID-19 epidemic by March 7.

[3] WHO-China Joint Mission (2020). Report of the WHO-China joint mission on coronavirus disease 2019 (COVID-19).

[4] Li J, Wang Y, Gilmour S, Wang M, Yoneoka D, Wang Y (2020). Estimation of the epidemic properties of the 2019 novel coronavirus: a mathematical modeling study.

[5] World Health Organization (2020). Report of the WHO-China joint mission on coronavirus disease 2019 (COVID-19).

[6] Word Health Organization (2020). Novel coronavirus (COVID-19) situation.

[7] Wu Z, McGoogan JM (2020). Characteristics of and important lessons from the coronavirus disease 2019 (COVID-19) outbreak in China: summary of a report of 72314 cases from the chinese center for disease control and prevention. JAMA..

[8] Services USDoHaH (1994). Public health in America.

[9] Donaldson L (2008). The UK public health system: change and constancy. Public Health.

[10] Tatara K, Okamoto E (2009). Japan: Health system review. Health Syst Transit.

[11] Yokoyama Y, Hirano K, Sato M, Abe A, Uebayashi M, Kishi E (2014). Activities and health status of dispatched public health nurses after the great East Japan earthquake. Public Health Nurs.

[12] Su TP, Lien TC, Yang CY, Su YL, Wang JH, Tsai SL (2007). Prevalence of psychiatric morbidity and psychological adaptation of the nurses in a structured SARS caring unit during outbreak: a prospective and periodic assessment study in Taiwan. J Psychiatr Res.

[13] Shiao JS, Koh D, Lo LH, Lim MK, Guo YL (2007). Factors predicting nurses’ consideration of leaving their job during the SARS outbreak. Nurs Ethics.

[14] Lung FW, Lu YC, Chang YY, Shu BC (2009). Mental symptoms in different health professionals during the SARS attack: a follow-up study. Psychiatr Q.

[15] McAlonan GM, Lee AM, Cheung V, Cheung C, Tsang KW, Sham PC (2007). Immediate and sustained psychological impact of an emerging infectious disease outbreak on health care workers. Can J Psychiatr.

[16] Lee AM, Wong JG, McAlonan GM, Cheung V, Cheung C, Sham PC (2007). Stress and psychological distress among SARS survivors 1 year after the outbreak. Can J Psychiatr.

[17] Chen NH, Wang PC, Hsieh MJ, Huang CC, Kao KC, Chen YH (2007). Impact of severe acute respiratory syndrome care on the general health status of healthcare workers in taiwan. Infect Control Hosp Epidemiol.

[18] Maunder RG, Lancee WJ, Balderson KE, Bennett JP, Borgundvaag B, Evans S (2006). Long-term psychological and occupational effects of providing hospital healthcare during SARS outbreak. Emerg Infect Dis.

[19] Marjanovic Z, Greenglass ER, Coffey S (2007). The relevance of psychosocial variables and working conditions in predicting nurses’ coping strategies during the SARS crisis: an online questionnaire survey. Int J Nurs Stud.

[20] Lee SH, Juang YY, Su YJ, Lee HL, Lin YH, Chao CC (2005). Facing SARS: psychological impacts on SARS team nurses and psychiatric services in a Taiwan general hospital. Gen Hosp Psychiatry.

[21] Lancee WJ, Maunder RG, Goldbloom DS (2008). Coauthors for the Impact of SS. Prevalence of psychiatric disorders among Toronto hospital workers one to two years after the SARS outbreak. Psychiatr Serv.

[22] Chan AO, Huak CY (2004). Psychological impact of the 2003 severe acute respiratory syndrome outbreak on health care workers in a medium size regional general hospital in Singapore. Occup Med.

[23] Khalid I, Khalid TJ, Qabajah MR, Barnard AG, Qushmaq IA (2016). Healthcare workers emotions, perceived stressors and coping strategies during a MERS-CoV outbreak. Clin Med Res.

[24] Alsahafi AJ, Cheng AC (2016). Knowledge, attitudes and behaviours of healthcare workers in the Kingdom of Saudi Arabia to MERS coronavirus and other emerging infectious diseases. Int J Environ Res Public Health.

[25] Abolfotouh MA, AlQarni AA, Al-Ghamdi SM, Salam M, Al-Assiri MH, Balkhy HH (2017). An assessment of the level of concern among hospital-based health-care workers regarding MERS outbreaks in Saudi Arabia. BMC Infect Dis.

[26] Wang L, Wang Z, Ma Q, Fang G, Yang J (2019). The development and reform of public health in China from 1949 to 2019. Glob Health.

[27] National Health Commission of the People’s Republic of China (2020). Guideline of COVID-19 prevention and control (fifith edition).

[28] Fayers PM, Sprangers MA (2002). Understanding self-rated health. Lancet.

[29] Lam CL, Tse EY, Gandek B (2005). Is the standard SF-12 health survey valid and equivalent for a Chinese population?. Qual Life Res.

[30] Wang W, Bian Q, Zhao Y, Li X, Wang W, Du J (2014). Reliability and validity of the Chinese version of the Patient Health Questionnaire (PHQ-9) in the general population. Gen Hosp Psychiatry.

[31] Spitzer RL, Kroenke K, Williams JB, Lowe B (2006). A brief measure for assessing generalized anxiety disorder: the GAD-7. Arch Intern Med.

[32] Hellewell J, Abbott S, Gimma A, Bosse NI, Jarvis CI, Russell TW (2020). Feasibility of controlling COVID-19 outbreaks by isolation of cases and contacts. Lancet Glob Health.

[33] Liu C, Liu S, Yang S, Wu H (2019). Association between transformational leadership and occupational burnout and the mediating effects of psychological empowerment in this relationship among CDC employees: a cross-sectional study. Psychol Res Behav Manag.

[34] Liu F (2017). Study on job burnout between nurses and pyisicians and its related factors.

[35] Team MHPaSD (2000). Mental health and work: impact, issues and good practices.

[36] Mihailescu M, Neiterman E (2019). A scoping review of the literature on the current mental health status of physicians and physicians-in-training in North America. BMC Public Health.

[37] Cone DC, Cummings BA (2006). Hospital disaster staffing: if you call, will they come?. Am J Disaster Med.

[38] Qureshi K, Gershon RR, Sherman MF, Straub T, Gebbie E, McCollum M (2005). Health care workers’ ability and willingness to report to duty during catastrophic disasters. J Urban Health.

[39] Nickell LA, Crighton EJ, Tracy CS, Al-Enazy H, Bolaji Y, Hanjrah S (2004). Psychosocial effects of SARS on hospital staff: survey of a large tertiary care institution. CMAJ..

[40] Kang HS, Son YD, Chae SM, Corte C (2018). Working experiences of nurses during the Middle East respiratory syndrome outbreak. Int J Nurs Pract.

