# Supplementary material for: Working conditions and health status of 6,317 front line public health workers across five provinces in China during the COVID-19 epidemic: a cross-sectional study
Source: BMC Public Health. 2021 Jan 9;21:106. doi: 10.1186/s12889-020-10146-0 (PMC7794632; doi:10.1186/s12889-020-10146-0)
Supplement: Supplementary file 1 — Additional file 1: Table S1. Work contents during the COVID-19 epidemic. Table S2. Areas of routine work of public health workers in Guangdong province during the COVID-19 epidemic. [file 12889_2020_10146_MOESM1_ESM.docx]

**Table S1 Work contents during the COVID-19 epidemic**

|  | All | | CDC workers | | PHI workers | | *p value* |
| --- | --- | --- | --- | --- | --- | --- | --- |
|  | *n* | *%* | *n* | *%* | *n* | *%* |  |
| **Field work** |  |  |  |  |  |  |  |
| Epidemiological investigation of the patients (face-to-face) | 1443 | 22.8 | 411 | 17.8 | 1032 | 25.8 | <0.001 |
| Epidemiological investigation of the patients (by phone/video) | 1310 | 20.7 | 616 | 26.6 | 694 | 17.3 | <0.001 |
| Epidemiological investigation of the close contacts (face-to-face) | 1495 | 23.7 | 441 | 19.1 | 1054 | 26.3 | <0.001 |
| Epidemiological investigation of the close contacts (by phone/video) | 1295 | 20.5 | 558 | 24.1 | 737 | 18.4 | <0.001 |
| Medical observation of the close contacts | 1440 | 22.8 | 236 | 10.2 | 1204 | 30.1 | <0.001 |
| On-site disinfection | 1427 | 22.6 | 469 | 20.3 | 958 | 23.9 | 0.001 |
| Specimen collection | 694 | 11.0 | 405 | 17.5 | 289 | 7.2 | <0.001 |
| Specimen shipment | 500 | 7.9 | 393 | 17.0 | 107 | 2.7 | <0.001 |
| Health education | 2618 | 41.4 | 526 | 22.7 | 2092 | 52.2 | <0.001 |
| Logistic support | 1322 | 20.9 | 486 | 21.0 | 836 | 20.9 | 0.901 |
| Management and coordination of the isolation sites | 822 | 13.0 | 195 | 8.4 | 627 | 15.7 | <0.001 |
| Supervision | 1213 | 19.2 | 522 | 22.6 | 691 | 17.3 | <0.001 |
| Community-based investigation | 249 | 3.9 | 15 | 0.6 | 234 | 5.8 | <0.001 |
| Fever detection and investigation at transportation hubs | 469 | 7.4 | 43 | 1.9 | 426 | 10.6 | <0.001 |
| **Non-field work** |  |  |  |  |  |  |  |
| Preparation of technical guidelines | 269 | 4.3 | 163 | 7.0 | 106 | 2.6 | <0.001 |
| Case reporting directly to national data center | 779 | 12.3 | 199 | 8.6 | 580 | 14.5 | <0.001 |
| Analysis and projection of the epidemic | 519 | 8.2 | 341 | 14.7 | 178 | 4.4 | <0.001 |
| Report writing | 962 | 15.2 | 601 | 26.0 | 361 | 9.0 | <0.001 |
| Laboratory test | 375 | 5.9 | 223 | 9.6 | 152 | 3.8 | <0.001 |
| Information correction and publicity | 1755 | 40.3 | 557 | 31.9 | 1198 | 45.9 | <0.001 |
| Technical training | 659 | 10.4 | 281 | 12.1 | 378 | 9.4 | 0.001 |
| Comprehensive coordination | 1547 | 24.5 | 592 | 25.6 | 955 | 23.9 | 0.121 |

**Table S2 Areas of routine work of public health workers in Guangdong province during the COVID-19 epidemic**

| Areas of routine work | All | | CDC workers | | PHI workers | | *p value* |
| --- | --- | --- | --- | --- | --- | --- | --- |
|  | *n* | *%* | *n* | *%* | *n* | *%* |  |
| **Public health concentration** | 1023 | 51.9 | 506 | 88.6 | 517 | 37.0 | <0.001 |
| Infectious disease prevention and control (e.g., STD/HIV/TB) | 134 | 6.8 | 92 | 16.1 | 42 | 3.0 |  |
| Non-communicable disease prevention and control | 403 | 20.5 | 115 | 20.1 | 288 | 20.6 |  |
| Immunization | 64 | 3.2 | 36 | 6.3 | 28 | 2.0 |  |
| Health education | 55 | 2.8 | 35 | 6.1 | 20 | 1.4 |  |
| Health inspection | 61 | 3.1 | 53 | 9.3 | 8 | 0.6 |  |
| Disinfection and prevention of vector-born diseases | 43 | 2.2 | 43 | 7.5 | 0 | 0 |  |
| Maternal and child health | 44 | 2.2 | 1 | 0.2 | 43 | 3.1 |  |
| Disease control/prevention (other diseases) | 62 | 3.1 | 50 | 8.8 | 12 | 0.9 |  |
| Others | 157 | 8.0 | 81 | 14.2 | 76 | 5.4 |  |
| **Non-public health concentration** | 947 | 48.1 | 65 | 11.4 | 882 | 63.0 | <0.001 |
| Clinicians | 419 | 21.3 | 33 | 5.8 | 386 | 27.6 |  |
| Nursing | 380 | 19.3 | 8 | 1.4 | 372 | 26.6 |  |
| Pharmacist | 72 | 3.7 | 0 | 0 | 72 | 5.1 |  |
| Clinical technician | 76 | 3.9 | 24 | 4.2 | 52 | 3.7 |  |
