# Supplementary material for: Working conditions and health status of 6,317 front line public health workers across five provinces in China during the COVID-19 epidemic: a cross-sectional study
Source: BMC Public Health. 2021 Jan 9;21:106. doi: 10.1186/s12889-020-10146-0 (PMC7794632; doi:10.1186/s12889-020-10146-0)
Supplement: Supplementary file 2 — Additional file 2. [file 12889_2020_10146_MOESM2_ESM.docx]

**Investigation of the "new coronary pneumonia epidemic" by public health control and prevention workers**

Dear Outbreak Prevention and Control Worker.

With the recent epidemic, first of all, I would like to express my most sincere thanks and respect for your efforts. Through this survey, we hope to document the status of public health workers on the epidemic control front, the time they spend, the work they do and the pressures they face. If you are currently involved in the public health prevention and control of the epidemic (such as prevention and control, health education, community public health work, etc., excluding clinical treatment, neighborhood committees, police work), we invite you to participate in the survey, we hope that the results will help more people know about the public health epidemic prevention and control work, and also hope that the results will provide a basis for the improvement of the epidemic prevention and control system. The survey is anonymous and voluntary and takes 8-10 minutes. All data collected will be analyzed only comprehensively and in absolute confidentiality. If you agree to participate, please start filling out the survey below. Again, hats off and thank you!

School of Public Health, Sun Yat-sen University

I. General information

1. Your gender: ①Male ②Female

2. Your age： ________________________________

3. How old is your youngest child? ①No children ②<1 year old ③1 to 2years old ④3 to 5 years old ⑤Elementary school students ⑥Junior high school students ⑧High school students ⑦University students and above

4. Type of unit you work for. _________________________________

5. Your current profession (please fill in the most important)_________________________________

6. Your job title: ① junior ② intermediate ③ associate ④ senior ⑤ other (e.g. volunteer, unspecified)

7. The city where your facility is located:__________________________

II. Epidemic prevention and control

8. What outbreak prevention and control field work are you currently involved in? [Multiple choice]

①None ②Patient transfer (face-to-face) ③Patient transfer (phone/video)④Interface transfer (face-to-face) ⑤Interface transfer (phone/video) ⑥Interface tracking management ⑦Interface medical observation ⑧Medical observation of infected area personnel ⑨On-site extermination ⑩Sample collection ⑪Sample transportation ⑫Health education ⑬On-site guidance ⑭Logistic support ⑮Hospitalization control ⑯Other (please add) _________________

9. What off-site work are you currently involved in outbreak prevention and control? [Multiple choice]

①None ②Preparation of technical guidelines ③Online reporting/revision ④Epidemic analysis ⑤Report writing ⑥Laboratory testing ⑦Information and publicity ⑧Other (please add) _________________

10. What organizations are you currently involved in coordinating
outbreak prevention and control? [Multiple choice]

①None ②Comprehensive coordination ③Information coordination

④Quarantine point management and coordination ⑤Technical training ⑥Supervision and inspection ⑦Other (please add) _________________

11. If you are currently involved in outbreak prevention and control efforts not covered above, please add here: 1) No additions 2) Yes additions. _________________

12. Is there a connection between the work done during the outbreak and the work done before your outbreak? ① not at all ② not very relevant ③ relevant ④ highly relevant ⑤ not sure

13. How many hours of outbreak-related training have you received? ①None②1-2 hours ③3-4 hours (half day) ④5-8 hours (1 day) ⑤1-2 days ⑥2 or more days

14. Do you feel that your knowledge of epidemic prevention and control is adequate? ①very adequate ②more adequate ③average ④less adequate ⑤very inadequate

15. Date your participation in outbreak prevention and control began._________________________________

16. Working days during the Spring Festival holiday (the 30th to the 6th

day of the Lunar New Year): ①0②1③2④3⑤4⑥5⑦6⑧7

17. Number of days of overnight work since joining the epidemic:①0②1③2④3⑤4⑥5⑦6⑧7⑨> 7

III. Health and Concerns

18. In the last week, how do you feel about your health: ①very good ②good ③fair ④poor ⑤very poor

19. Please select the level that is most appropriate for you for the following situations.

|  | Extremely light | Lighter | Moderate | Relatively high | Extremely high |
| --- | --- | --- | --- | --- | --- |
| Worried about the extent of their infection at work | ① | ② | ③ | ④ | ⑤ |

20. Do you have any of the following practices to avoid influencing your family yourself? [Multiple choice] ①Disinfecting the entire body before going home②Wearing a mask at home③Living in a separate room④Not living at home⑤Sending young children to parents' home⑥Reducing close contact with family⑦Living alone, not living with family⑧Other (please add) _________________⑨None of the above.

IV. Working conditions

21. Have you received much support in your work on the epidemic?

|  | None | Very few | Moderate | Many | A great deal |
| --- | --- | --- | --- | --- | --- |
| 1) Support from colleagues | ① | ② | ③ | ④ | ⑤ |
| 2) Support from family members | ① | ② | ③ | ④ | ⑤ |
| 3) Support from society | ① | ② | ③ | ④ | ⑤ |

22. Do you have any of the following problems with the epidemic work?

|  | None | very few | moderate | many | A great deal |
| --- | --- | --- | --- | --- | --- |
| 1) Your work is sometimes not understood | ① | ② | ③ | ④ | ⑤ |
| 2) Unfair treatment at work | ① | ② | ③ | ④ | ⑤ |
| 3) Sometimes you feel aggrieved at work | ① | ② | ③ | ④ | ⑤ |
| 4) Your family doesn't understand your efforts | ① | ② | ③ | ④ | ⑤ |
| 5) Concerns about routine work pressures beyond the epidemic | ① | ② | ③ | ④ | ⑤ |

V. Perceptions of the epidemic

23. How long do you think you can continue in your current job? ①1-2 weeks②2-3 weeks ③3-4 weeks ④1-2 months ⑤2-3 months ⑥>3 months

VI. Anxiety and depression

24. There are also two final scales that relate to your recent anxiety and depression. The standardized questions give more reliable and comparable results, but if you find them too long to fill out, we understand and you can click "skip". Thank you for your support! ① Skip (Please skip to question 46) ② Fill in the blanks.

25. How often have you been troubled by the following questions in the past two weeks?

|  | Not at all | In less than half the time. | More than half the time. | Almost every day |
| --- | --- | --- | --- | --- |
| 1) Feeling nervous, restless or irritable | ① | ② | ③ | ④ |
| 2) Inability to stop or control worries | ① | ② | ③ | ④ |
| 3) Worrying excessively about different things | ① | ② | ③ | ④ |
| 4) Difficult to relax | ① | ② | ③ | ④ |
| 5) Restlessness due to a lack of peace of mind | ① | ② | ③ | ④ |
| 6) Easily upset or irritable | ① | ② | ③ | ④ |
| 7) Feeling scared and thinking something terrible is going to happen | ① | ② | ③ | ④ |

26. How often have you been troubled by the following questions in the

past two weeks?

|  | Not at all | In less than half the time. | More than half the time. | Almost every day |
| --- | --- | --- | --- | --- |
| 1) Lack of motivation or pleasure in doing things | ① | ② | ③ | ④ |
| 2) Feeling depressed, frustrated or hopeless | ① | ② | ③ | ④ |
| 3) Difficulty falling asleep, restless sleep, or excessive sleeping | ① | ② | ③ | ④ |
| 4) Feeling tired or lacking energy | ① | ② | ③ | ④ |
| 5) Loss of appetite or eating too much | ① | ② | ③ | ④ |
| 6) Feeling like shit, or a failure, or letting yourself or your family down | ① | ② | ③ | ④ |
| 7) Difficulty  concentrating on  things, such as when  reading a newspaper  or watching  television | ① | ② | ③ | ④ |
| 8) moves or speaks  so slowly that others  are already aware of  it, or just the  opposite - more than  usual fidgeting  or fidgeting and  moving around | ① | ② | ③ | ④ |
| 9) Having thoughts  that are better than  dying or hurting  yourself in some way | ① | ② | ③ | ④ |
